# Exogenous indole-3-acetic acid promotes the plant growth and accumulation of selenium in grapevine under selenium stress

**DOI:** 10.1186/s12870-024-05105-5

**Published:** 2024-05-20

**Authors:** Jin Wang, Lei Liu, Haiyan Zhang, Dilian Zhang, Zhen Dai, Xian Luo, Xiaoli Zhang, Hui Xia, Dong Liang, Xiulan Lv, Lijin Lin

**Affiliations:** 1 College of Horticulture, Sichuan Agricultural University, Chengdu, Sichuan China; 2 Institute of Horticulture Research, Chengdu Academy of Agriculture and Forestry Sciences, Chengdu, China

**Keywords:** IAA, Selenium stress, Grape, Growth, Stress physiology

## Abstract

To alleviate the selenium (Se) stress in fruit trees and improve its accumulation, the effects of exogenous indole-3-acetic acid (IAA) on the growth and Se accumulation of grapevine under Se stress were studied. The application of exogenous IAA increased the biomass of grapevine, and the concentration of exogenous IAA had a regression relationship with the biomass. The root and shoot biomass were the maximum at 60 mg L^− 1^ IAA, increasing by 15.61% and 23.95%, respectively, compared with the control. Exogenous IAA also increased the photosynthetic pigments and the activities of superoxide dismutase and peroxidase in grapevine. Moreover, exogenous IAA increased the contents of total Se, organic Se, and inorganic Se, and the concentration of exogenous IAA had a regression relationship with the total Se content. The highest contents of root total Se and shoot total Se were accumulated at 90 mg L^− 1^ IAA, increasing by 29.94% and 55.77% respectively,. In addition, the correlation and path analyses revealed that the carotenoid content and root total Se content were closely associated with the shoot total Se content. Therefore, the application of exogenous IAA can alleviate the stress of Se to grape and promote its uptake and the most effective amount for the uptake of Se is 90 mg L^− 1^ IAA.

**Supplementary Information:**

The online version contains supplementary material available at 10.1186/s12870-024-05105-5.

## Introduction

Selenium (Se) participates in major metabolic processes necessary for cellular metabolism in human bodies [1–3]. Humans can obtain Se from food, and the consumption of crops is the primary source [4]. Thus, improving the amount of Se in crops can improve its supply for humans. However, Se concentrations in soils vary widely across the globe, typically ranging from 0.01 to 2.00 mg kg^− 1^, with an average soil Se concentration of 0.4 mg kg^− 1^, which results in a low Se content in crops [3]. Application of low doses of Se fertilizers on crops can promote their growth [5], but its constant use or the over-application for biofortification greatly can promote the release of Se into agroecosystems, with potentially toxic effects on crops [6]. Se stress can also cause stunted growth, chlorosis and crop death [7, 8]. Thus, it is necessary to determine measures for crops that improve their tolerance to Se stress.

There are some measures that can improve the tolerance of crops to Se stress, including intercropping, grafting and phytohormones [9–11]. Among these measures, some phytohormones can improve the tolerance of plants to abiotic stress [12]. Auxin is a key phytohormone that regulates plant growth and development, and it is primarily found in the form of indole-3-acetic acid (IAA) [13]. IAA can regulate plant morphogenesis, organ development, and aptitude response and tissue differentiation throughout the whole growth of plants [14, 15]. In the cultivation of fruit trees, IAA has been found to promote the rooting of cuttings, enhance tolerance to stress conditions and regulate the secondary metabolism of fruit [16–18]. Under low conditions of Se, this compound can stimulate the elongation of the primary roots and the number of lateral roots by the biosynthesis and transport of IAA in tobacco [19]. Another study also shows that the levels of expression of the IAA biosynthetic genes (*NtYUCCA*) and polar transport protein genes (*NtPINs*) in tobacco are up-regulated at low concentrations of Se and promote the accumulation of IAA in the roots, but the plants react in an opposite manner at high concentrations of Se [20]. However, treatment with Se suppresses the levels of expression of the genes related to IAA in *Arabidopsis thaliana* [21]. The above studies suggest that IAA accumulation in plants may be regulated by the concentration of Se in the environment, and IAA may be involved as a signaling molecule in the response of plants to Se. In addition, the application of exogenous IAA alleviates inhibitory effects on the length of growth and number of lateral roots of rice under Se stress [22]. In maize, the addition of exogenous IAA not only regulates the uptake of sodium (Na^+^), potassium (K^+^), and calcium (Ca^2+^), but also increases the Se content in maize, thereby altering the accumulation and distribution of Se [23]. Exogenous IAA also alleviates Se stress in tamarillo seedlings and increases their Se content [11]. Thus, IAA may promote the accumulation of Se in crops, but to our knowledge, there have been no reports about its effects on fruit trees.

Grape (*Vitis vinifera* L.) is one of the four major fruits in the world, and it is highly nutritious and economically valuable [24]. Abiotic stress limits the growth, yield and quality of grapes [25, 26]. A previous study shows that the growth of grapevine is inhibited by an aqueous solution of 0.10 mg L^− 1^ Se (in the form of Na_2_SeO_3_) [26]. If IAA is applied on grape, its tolerance to Se and the accumulation of Se can be improved. Therefore, an investigation to determine the effects of exogenous IAA on the growth and Se accumulation of grapevine under Se stress was conducted. Another objective of this experiment was to determine the best concentration of IAA that could alleviate the Se stress and promote the Se uptake in grapes.

## Materials and methods

### Materials

The grape variety was ‘Summer Black’ grape. Stem cuttings of the grape were collected from the vineyard of Sichuan Agricultural University in December 2019. The collected stem cuttings were buried in moist sand for storage. In February 2020, 10 cm-long sections of the stem were cut with one bud and placed in a tray filled with moist perlite. The tray was placed in the conditions of 14 h, 10,000 lx, 25 ºC and relative humidity 70% during the day and 10 h, 0 lx, 20 °C and relative humidity 90% at night [27]. The stem cuttings were irrigated with one-half Hoagland solution every 3 d until the grape roots grew out, and the new grape shoots had grown to 15–16 cm.

IAA was obtained from Beijing Solarbio Science & Technology Co., Ltd., Beijing, China.

Hoagland solution includes potassium nitrate 607 mg L^− 1^, ammonium phosphate 115 mg L^− 1^, magnesium sulfate 493 mg L^− 1^, iron salt solution 2.5 mL L^− 1^, trace elements 5 mL L^− 1^ and pH = 6.0. iron salt solution includes ferrous sulfate heptahydrate 2.78 g, disodium ethylenediaminetetraacetate (EDTA) 3.73 g, distilled water 500 mL, and pH = 5.5. Trace element solution includes potassium iodide 0.83 mg L^− 1^, boric acid 6.2 mg L^− 1^, manganese sulfate 22.3 mg L^− 1^, zinc sulfate 8.6 mg L^− 1^, sodium molybdate 0.25 mg L^− 1^, copper sulfate 0.025 mg L^− 1^ and cobalt chloride 0.025 mg L^− 1^.

### Experimental design

In March 2020, uniform grape seedlings (five leaves) were transplanted into the plastic pots (15 cm in height × 18 cm in diameter) that were filled with perlite. Two grape seedlings were planted in each pot, and the pots were placed in the same conditions used to raise the grapevine nursery. A volume of 100 mL Hoagland solution that contained 0.1 mg L^− 1^ Se (in the form of Na_2_SeO_3_) [27] was added to each pot every 3 d until harvesting. Then, IAA solutions at concentrations of 0, 30, 60, 90 and 120 mg L^− 1^ were applied on the grape seedlings (Table 1) [16–18]. IAA solutions were sprayed on both sides of the leaves until droplets were formed at the leaf tips that were going to drop (about 10 mL for each pot, and about 60 mL in total for each IAA concentration). The experimental design was completely randomized design, and each treatment was repeated three times with two pots as a replicate (30 pots in total). The seedlings were sprayed with IAA again after 15 d.

**Table 1:** Experimental treatments

Treatments	IAA concentration (mg L^− 1^)				
	0	30	60	90	120
Se concentration (mg L^− 1^)	0.10	0.10	0.10	0.10	0.10

### Determination of Se and physiological parameters

One month after the first treatment with IAA (April 2020), the mature leaves of grapevine were collected to determine the contents of photosynthetic pigments (chlorophyll *a*, chlorophyll *b*, and carotenoid) and the activities of antioxidant enzymes [superoxide dismutase (SOD), peroxidase (POD), and catalase (CAT)]. The photosynthetic pigments were used the acetone and ethanol (1: 1) extraction method [28] to determine. The activities of SOD, POD, and CAT were assayed using the nitroblue tetrazolium photoreduction, guaiacol colorimetry and UV spectrophotometry methods, respectively, according to Lin et al. (2020) [29] and Hao et al. (2004) [28]. After that, the whole grapevines were dug up and washed. The grapevines were divided into roots and shoots and dried to determine their biomass (dry weight) [30]. The dried samples were finely ground, and digested with 4:1 (v/v) nitrate: perchloric acid and reduced with HCl. The digested solutions were used to determine the content of Se using hydride generation-atomic fluorescence spectrometry (AFS-9700; Beijing Haiguang Instrument Co., Ltd., Beijing, China) [31]. Another finely ground samples were extracted with 6 mol L^− 1^ HCl, and the extracted solution was used to determine the content of inorganic Se using hydride generation-atomic fluorescence spectrometry, and the content of organic Se was calculated as the content of total Se minus inorganic Se [30]. The translocation factor (TF) of Se was calculated as the shoot Se content/root Se content [32].

### Statistical analysis

All the data were analyzed using SPSS 26.0 (IBM, Inc., Armonk, NY, USA). All the data were normalized and tested for homogeneity using a one-way analysis of variance (ANOVA) and Duncan’s Multiple Range Test (*p <* 0.05). The relationship between the concentration of IAA and biomass or total Se content was analyzed by a regression analysis. A Pearson’s correlation was used to calculate the correlation among all the indicators (0.01 ≤ *p* < 0.05 or *p* < 0.01). The path analysis was used to analyze the contributions of different indicators to the shoot total Se content [33].

## Results

### Grapevine biomass

**Fig. 1:**
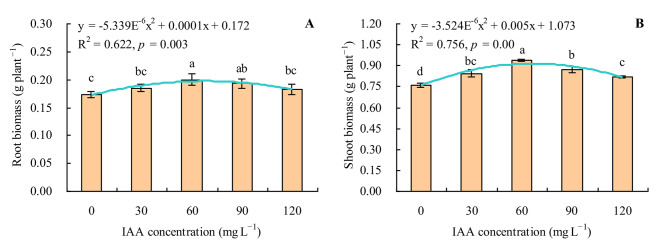
Biomass (dry weight) of grapevine. **A**: root biomass; **B**: shoot biomass. Values are means ± SD of three replicates. Different lowercase letters indicate significant differences among the treatments (Duncan’s Multiple Range Test, *p <* 0.05)

The biomass of grapevine was increased by exogenous IAA under Se stress (Fig. 1A and B). IAA concentration had a quadratic polynomial regression relationship with both the root and shoot biomass. Both the root and shoot biomass increased along with the increase of IAA concentration when ≤ 60 mg L^− 1^ but decreased when > 60 mg L^− 1^. Treatment with 60 and 90 mg L^− 1^ IAA increased the root biomass by 15.61% and 11.56% compared with the control respectively, whereas the treatment with 30 and 120 mg L^− 1^ IAA had no significant effects. Compared with the control, IAA at 30, 60, 90 and 120 mg L^− 1^ increased the shoot biomass of grapevine by 11.18%, 23.95%, 14.61% and 7.63% respectively.

### Contents of photosynthetic pigments in the grapevine leaves

The contents of chlorophyll *a* and carotenoid in grapevine leaves were increased by the different concentrations of exogenous IAA under Se stress (Table 2). Compared with the control, IAA at 30, 60, 90 and 120 mg L^− 1^ increased the content of chlorophyll *a* by 14.06%, 18.44%, 15.02% and 14.12% respectively, and increased the content of carotenoid by 12.58%, 40.18%, 36.50% and 28.83% respectively. IAA at 60 and 90 mg L^− 1^ increased the content of chlorophyll *b* under Se stress, while IAA at 30 and 120 mg L^− 1^ had no significant effects on this parameter.

**Table 2:** Photosynthetic pigment content in grapevine leaves

IAA concentration (mg L^− 1^)	Chlorophyll a content (mg g^− 1^ FW)	Chlorophyll b content (mg g^− 1^ FW)	Carotenoid content (mg g^− 1^ FW)
0	1.551 ± 0.040b	0.499 ± 0.029c	0.326 ± 0.010d
30	1.769 ± 0.058a	0.505 ± 0.022c	0.367 ± 0.002c
60	1.837 ± 0.056a	0.634 ± 0.026a	0.457 ± 0.012a
90	1.784 ± 0.067a	0.585 ± 0.020b	0.445 ± 0.006a
120	1.770 ± 0.064a	0.474 ± 0.012c	0.420 ± 0.006b

Values are means ± SD of three replicates. Different lowercase letters indicate significant differences among the treatments (Duncan’s Multiple Range Test, *p <* 0.05). FW = fresh weight

### Antioxidant enzyme activity of the grapevine leaves

Under Se stress, all concentrations of exogenous IAA increased the activities of SOD and POD in grapevine leaves, but had no significant effects on the activity of CAT (Table 3). IAA at 30, 60, 90 and 120 mg L^− 1^ increased the activity of SOD by 8.98%, 32.08%, 25.01% and 20.11%, respectively, and increased that of POD by 39.21%, 64.97%, 47.13% and 43.08%, respectively, compared with the control.

**Table 3:** Antioxidant enzyme activity of grapevine leaves

IAA concentration (mg L^− 1^)	SOD activity (U g^− 1^ FW)	POD activity (U g^− 1^ min^− 1^ FW)	CAT activity (mg g^− 1^ FW min^− 1^)
0	36.63 ± 0.74e	33.21 ± 0.73d	1.724 ± 0.043a
30	39.91 ± 0.64d	46.23 ± 0.28c	1.736 ± 0.014a
60	48.37 ± 0.79a	54.79 ± 1.05a	1.790 ± 0.019a
90	45.79 ± 0.87b	48.86 ± 0.40b	1.764 ± 0.061a
120	43.99 ± 0.53c	47.52 ± 0.82c	1.756 ± 0.036a

Values are means ± SD of three replicates. Different lowercase letters indicate significant differences among the treatments (Duncan’s Multiple Range Test, *p <* 0.05). FW = fresh weight

### Different forms of Se and their transport in grapevine

**Fig. 2:**
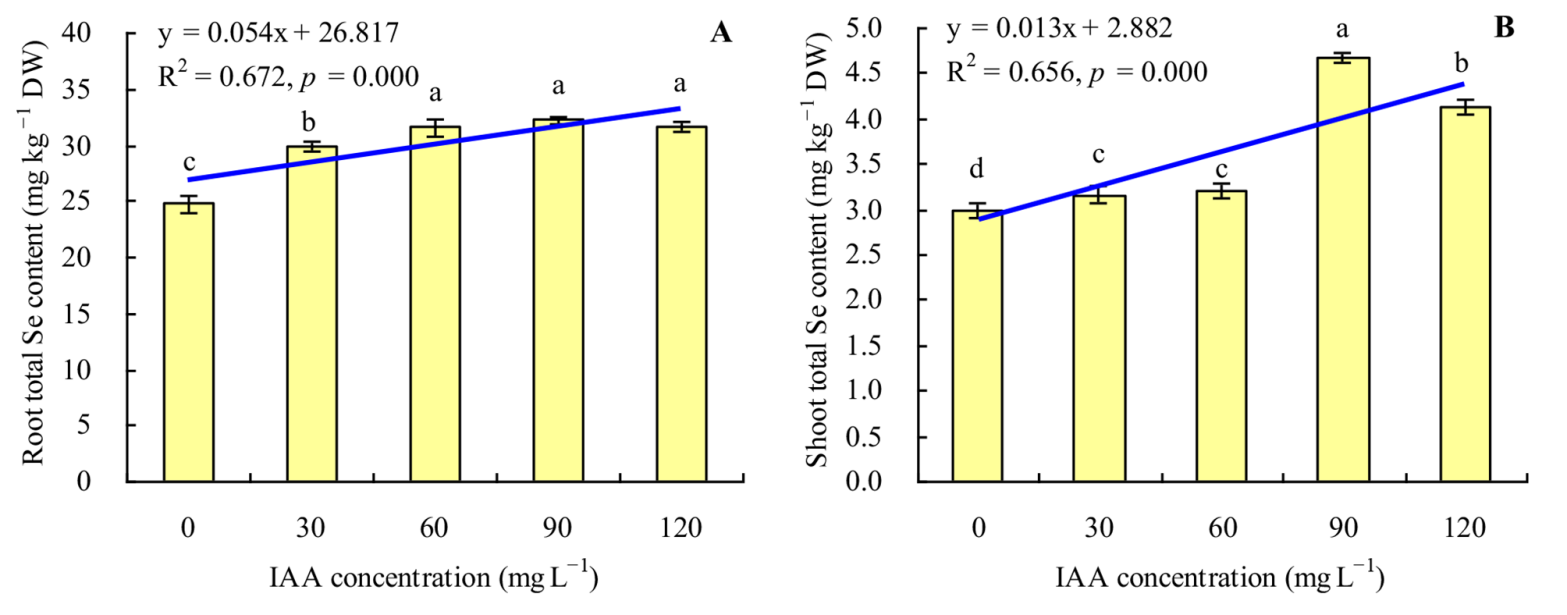
Total Se content in grapevine. **A**: root total Se content; **B**: shoot total Se content. Values are means ± SD of three replicates. Different lowercase letters indicate significant differences among the treatments (Duncan’s Multiple Range Test, *p <* 0.05). DW = dry weight

There was a much higher total Se content in roots of grapevine than in shoots (Fig. 2A and B). The total Se contents in various organs of grapevine were increased by all concentrations of exogenous IAA. The concentration of exogenous IAA had a positive linear regression with the total Se contents in both roots and shoots. Compared with the control, IAA at 90 mg L^− 1^ promoted the maximum accumulations of total Se in both root and shoot, increasing by 29.94% 55.77% respectively.

**Fig. 3:**
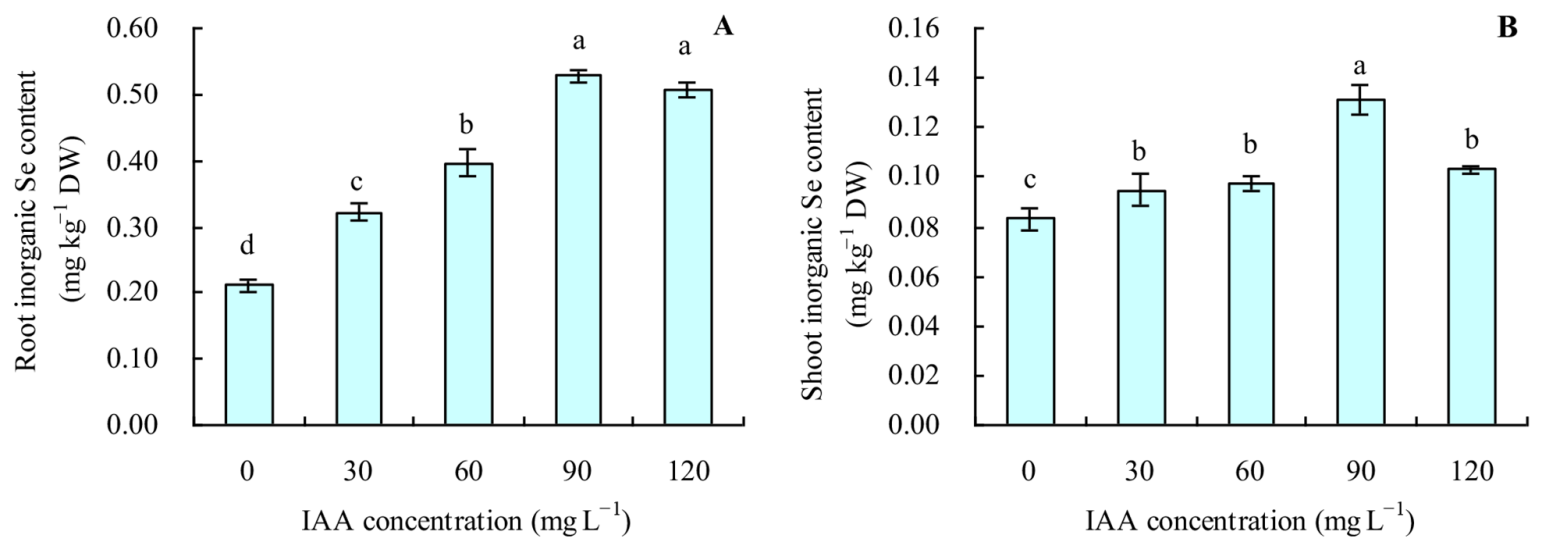
Inorganic Se content in grapevine. **A**: root inorganic Se content; **B**: shoot inorganic Se content. Values are means (± SD) of three replicates. Different letters indicate significant differences among the treatments (Duncan’s Multiple Range Test, *p* < 0.05). DW = dry weight

**Fig. 4:**
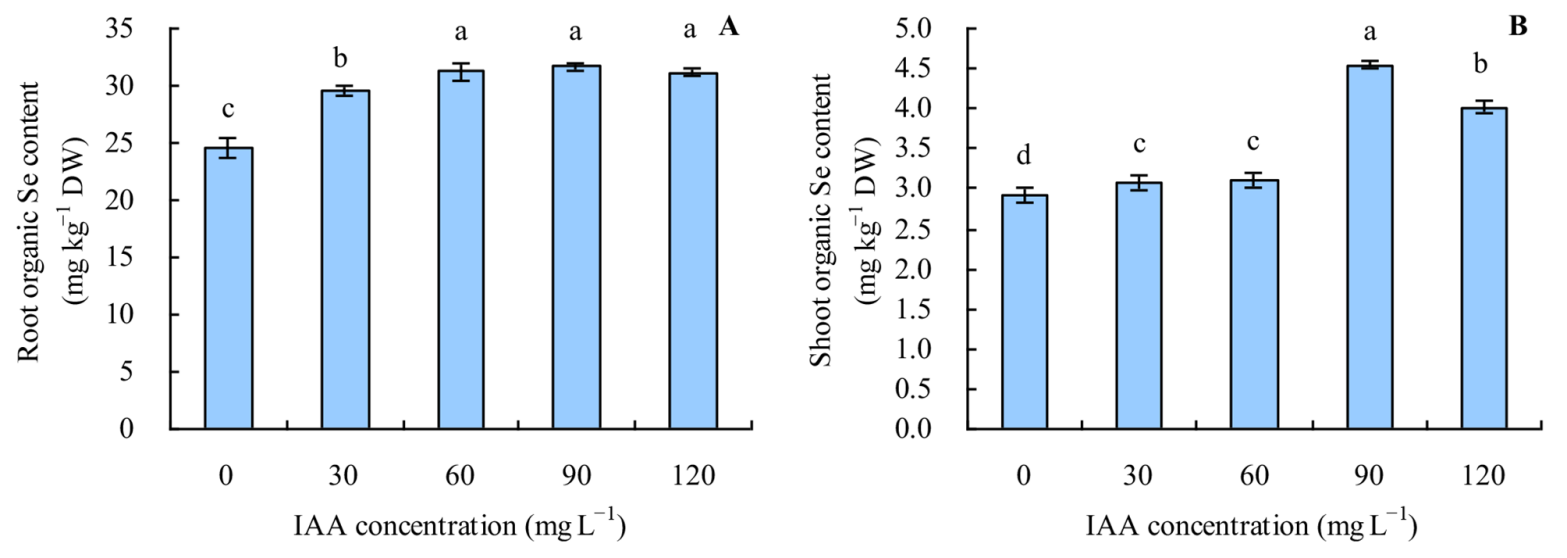
Organic Se content in grapevine. **A**: root organic Se content; **B**: shoot organic Se content. Values are means (± SD) of three replicates. Different letters indicate significant differences among the treatments (Duncan’s Multiple Range Test, *p* < 0.05). DW = dry weight

**Fig. 5:**
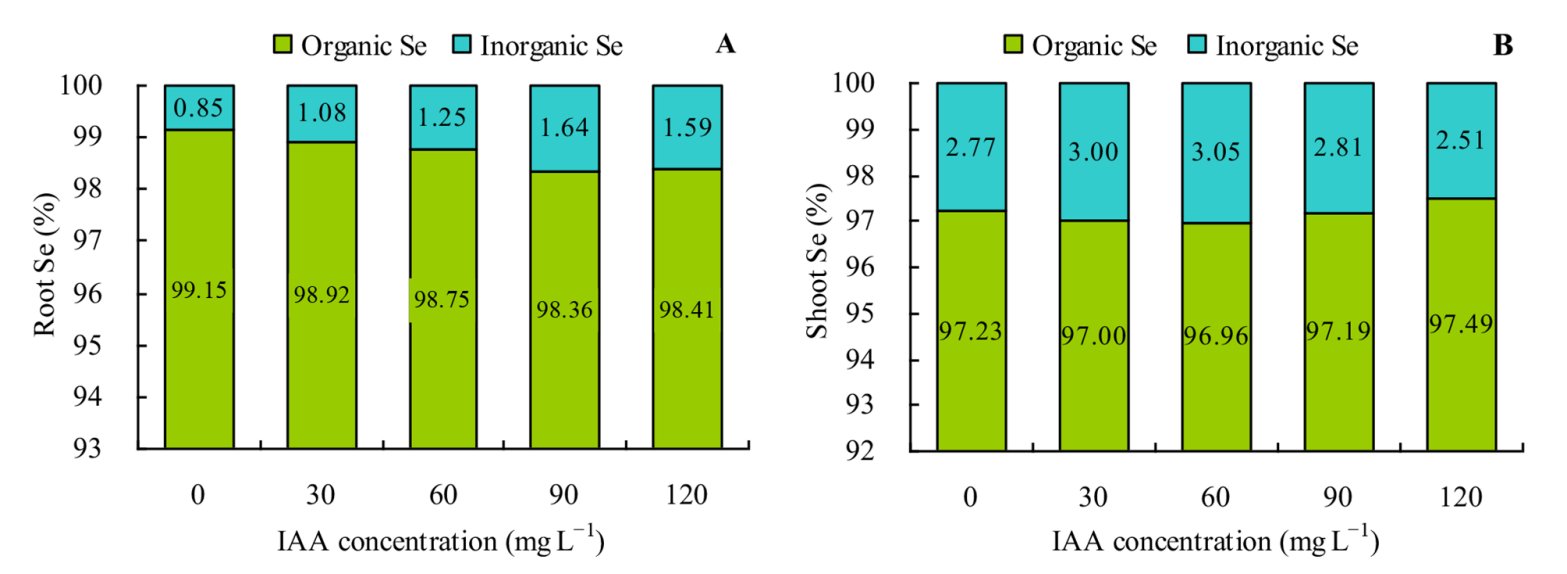
Ratios of organic Se and inorganic Se to total Se in grapevine. **A**: ratios of organic Se and inorganic Se to total Se to total Se in roots; **B**: ratios of organic Se and inorganic Se to total Se to total Se in shoots. Values are means ± SD of three replicates. Different lowercase letters indicate significant differences among the treatments (Duncan’s Multiple Range Test, *p <* 0.05)

The contents of inorganic Se and organic Se in various organs of grapevine were also increased by the different concentrations of exogenous IAA (Figs. 3A and B and 4A and B). With the increase in concentration of exogenous IAA, the contents of inorganic Se and organic Se in various organs changed in the same manner as the total Se content. The ratio of organic Se to total Se was > 95% in roots and shoots of grapevine. All concentrations of exogenous IAA decreased the ratio of organic Se and increased the ratio of inorganic Se to total Se in roots, while it had no apparent effects on the ratios of organic Se and inorganic Se to total Se in shoots (Fig. 5A and B).

**Fig. 6:**
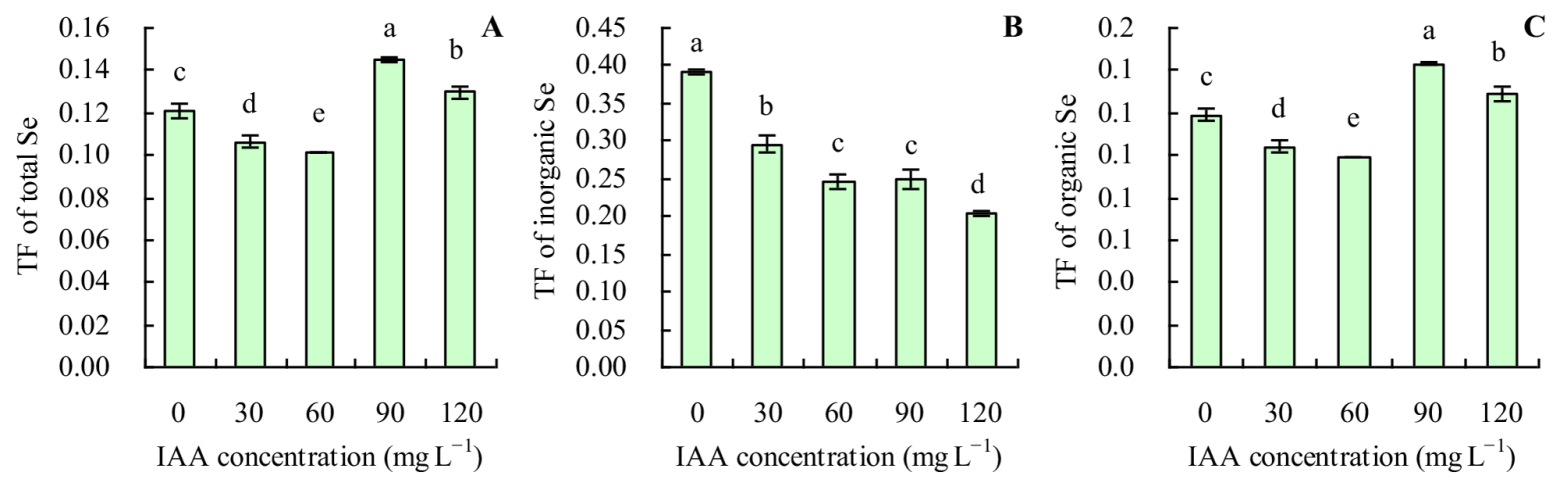
TFs of grapevine. **A**: TF of total Se; **B**: TF of inorganic Se; **C**: TF of organic. Values are means ± SD of three replicates. Different lowercase letters indicate significant differences among the treatments (Duncan’s Multiple Range Test, *p <* 0.05). TF = shoot Se content/ root Se content

IAA at 30 and 60 mg L^− 1^ decreased the TFs of total Se and organic Se, while IAA at 90 and 120 mg L^− 1^ increased these indicators (Fig. 6A and C). The order of the TFs of total Se and organic Se was 90 mg L^− 1^ IAA > 120 mg L^− 1^ IAA > 0 mg L^− 1^ IAA > 30 mg L^− 1^ IAA > 60 mg L^− 1^ IAA. However, the different concentrations of exogenous IAA decreased the TF of inorganic Se (Fig. 6B).

### Correlation and path analyses

A correlation analysis was used to examine the relationships of total Se content with the other parameters (Table 4). The root total Se content had a highly significant (*p* < 0.01) positive correlation with the root and shoot biomass, contents of chlorophyll *a* and carotenoid, and the activities of SOD and POD. The shoot total Se content had a significant (0.01 ≤ *p* < 0.05) positive correlation with the content of carotenoid and a highly significant (*p* < 0.01) positive correlation with the root total Se content.

**Table 4:** Correlations among the indicators

Indicator	Root biomass	Shoot biomass	Chlorophyll a content	Chlorophyll b content	Carotenoid content	SOD activity	POD activity	CAT activity	Root total Se content	Shoot total Se content
Root biomass	1									
Shoot biomass	0.746**	1								
Chlorophyll *a* content	0.575*	0.822**	1							
Chlorophyll *b* content	0.607*	0.824**	0.499	1						
Carotenoid content	0.727**	0.792**	0.779**	0.657**	1					
SOD activity	0.774**	0.847**	0.788**	0.705**	0.983**	1				
POD activity	0.739**	0.896**	0.904**	0.615*	0.904**	0.920**	1			
CAT activity	0.290	0.538*	0.520*	0.434	0.579*	0.534*	0.516*	1		
Root total Se content	0.654**	0.701**	0.861**	0.416	0.908**	0.867**	0.911**	0.486	1	
Shoot total Se content	0.206	0.121	0.343	0.086	0.587*	0.475	0.341	0.218	0.644**	1

**: Correlation is significant at the 0.01 level (2-tailed test). *: Correlation is significant at the 0.05 level (2-tailed test). *N* = 15

To further analyze the contributions of different indicators to the shoot total Se content, a path analysis was used in this study (Table 5). The direct path coefficients (absolute) of the carotenoid content, POD activity and of root total Se content were the top 3 largest indicators, which indicated that these three indicators directly affected the shoot total Se content. In addition, the top 3 largest indirect path coefficients (absolute) were the carotenoid content, SOD activity and POD activity, which indicated their indirect effects on the shoot total Se content. Moreover, the top 3 largest total path effect coefficients were the carotenoid content, SOD activity and root total Se content, which indicated that they had the largest contributions to the shoot total Se content.

**Table 5:** Path coefficients of the different indicators with the shoot total Se content

Factor	Total effect coefficient	Direct effect coefficient	Indirect effect coefficient									
			Total	X1→Y	X2→Y	X3→Y	X4→Y	X5→Y	X6→Y	X7→Y	X8→Y	X9→Y
X1	0.206	-0.236	0.442		0.427	-0.016	-0.089	1.375	-0.614	-1.496	-0.076	0.931
X2	0.120	0.572	-0.452	-0.176		-0.023	-0.121	1.497	-0.672	-1.814	-0.141	0.998
X3	0.343	-0.028	0.371	-0.135	0.470		-0.073	1.472	-0.626	-1.828	-0.136	1.227
X4	0.085	-0.147	0.232	-0.143	0.471	-0.014		1.242	-0.559	-1.244	-0.114	0.593
X5	0.587	1.891	-1.304	-0.171	0.453	-0.021	-0.097		-0.781	-1.828	-0.152	1.293
X6	0.475	-0.794	1.269	-0.182	0.485	-0.022	-0.104	1.859		-1.862	-0.140	1.235
X7	0.340	-2.023	2.363	-0.174	0.513	-0.025	-0.091	1.709	-0.731		-0.135	1.297
X8	0.218	-0.262	0.480	-0.068	0.308	-0.014	-0.064	1.095	-0.424	-1.044		0.691
X9	0.644	1.424	-0.780	-0.154	0.401	-0.024	-0.061	1.717	-0.689	-1.843	-0.127	

X1 = root biomass; X2 = shoot biomass; X3 = chlorophyll *a* content; X4 = chlorophyll *b* content; X5 = carotenoid content; X6 = SOD activity; X7 = POD activity; X8 = CAT activity; X9 = root total Se content; Y = shoot total Se content. Total path effect coefficient = direct path coefficient + indirect path coefficient

## Discussion

IAA plays an important role in the entire growth cycle of plants [14, 15]. A low level of Se can up-regulate the levels of expression of the IAA biosynthetic genes and promote the accumulation of IAA, while a high level of Se inhibits the levels of expression of the genes related to IAA in plants [19–21]. The application of IAA alleviates the inhibitory effect of Se stress on the growth of lateral roots in rice [22] and increases the biomass of tomatillo seedlings under Se stress [11], which indicates that exogenous IAA can regulate the growth of plants under Se stress. In this study, the application of exogenous IAA increased grapevine biomass under Se stress, and the IAA concentration exhibited a regression relationship with the biomass. These results indicated that exogenous IAA can alleviate Se stress and promote the growth of grapevine under Se stress, which were consistent with the results of previous studies [11, 22]. IAA can promote the growth of lateral roots and root hairs in plants, and enhance the area of root absorption [34], which is the possible reason for the increases of both root and shoot biomass in this research.

Chlorophyll biosynthesis is inhibited when the plants are subjected to stress, which may inhibit the photosynthesis of plants [35]. Exogenous IAA increases the net photosynthetic rate and accumulation of photosynthetic products [36]. The application of exogenous IAA increases the contents of photosynthetic pigments in tomatillo seedlings under Se stress [11]. In this experiment, exogenous IAA increased the contents of chlorophyll *a* and carotenoid in grapevine leaves, and only exogenous IAA at 60 and 90 mg L^− 1^ increased the content of chlorophyll *b* under Se stress. These results are consistent with the findings of a previous study [11], which indicates that exogenous IAA can promote the biosynthesis of photosynthetic pigments in grapevine under Se stress. The reason may be related to the ability of IAA promoting the photosynthetic pigments biosynthesis [37]. Under stress conditions, reactive oxygen species (ROS) rapidly accumulate in plants, and damage the cell membrane system. Antioxidant enzymes play an important role in removing this ROS [38]. Under drought conditions, the application of exogenous IAA increases the activities of SOD, POD and CAT in white clover (*Trifolium repens* L.) [39]. Contrarily, in the study on maize, exogenous IAA decreased the activities of SOD, POD and CAT under cadmium stress [40]. Thus, different effects of exogenous IAA may be produced under variable stress conditions. In this study, exogenous IAA increased the activities SOD and POD in grapevine, which indicated that exogenous IAA can improve the tolerance of grapevine to Se stress. These results were consistent with the previous studies [11, 39], which might be explained by the ability of IAA regulating the ascorbic acid-glutathione cycle [41]. However, exogenous IAA had no significant effect on the CAT activity of grapevine in this study. The possible reason may be that CAT is the most sensitive antioxidant enzyme to abiotic stress and its activity is firstly inhibited by abiotic stress, and the accumulated H_2_O_2_ is removed by POD, leading to an increase in POD activity [42, 43].

The distribution of Se in plants varies considerably in different valence states. Selenate is up-taken by plants is transported to the aboveground parts, whereas the form of selenate up-taken by plants is chiefly concentrated in their roots [44]. Most of the selenite in plant roots is directly assimilated into organic Se compounds, and only a small portion of the Se is transferred to other parts in the form of inorganic ions [45]. In this study, the root total Se content in grapevine was much higher than the shoot total Se content after sodium selenite treatments, and the ratio of organic Se to total Se was > 95% in both roots and shoots of grapevine. These results are consistent with those of previous studies [44, 45], which indicated that only a small portion of Se is transferred from the roots to shoots, and most of the Se is in the form of organic Se. In maize, under Se stress, exogenous IAA increased the content of Se, and altered its accumulation and distribution [23]. Under Se stress, treatment with exogenous IAA also increased the accumulation of Se in tomatillo seedlings [11]. In this study, exogenous IAA increased the contents of total Se, organic Se and inorganic Se in grapevine under Se stress, and the IAA concentration had a regression relationship with the total Se content. This indicated that treatment with exogenous IAA could promote the uptake of Se in grapevine. These results are the same as those in previous studies [11, 23] and further suggest that treatment with exogenous IAA can improve the tolerance of grapevine to Se stress and alleviate this stress in grapevine. The reason may be owing to the increase in antioxidants and the enhancement of sulfur assimilation [46], which merits further study. In addition, correlation and path analyses showed that the carotenoid content and root total Se content were closely associated with the shoot total Se content, which highlights their significant role in promoting the uptake of Se in grapevine under Se stress. Although this study just investigated the promotion effect of Se in grapevine, the Se accumulation in berry fruits of grape can be also increased by IAA according to the previous studies [47, 48]. The promotion effect of IAA on the Se uptake in berry fruits of grape need to be further studied.

## Conclusion

Exogenous IAA increased the biomass, contents of photosynthetic pigments, and the activities of SOD and POD of grapevine under Se stress. The concentration of exogenous IAA had a regression relationship with both the biomass. Exogenous IAA also increased the contents of total Se, organic Se, and inorganic Se in grapevine. The concentration of exogenous IAA had a regression relationship with the total Se content. IAA at 90 mg L^− 1^ produced the maximal amount of shoot total Se. In addition, correlation and path analyses revealed that the carotenoid content and root total Se content were closely associated with the shoot total Se content. Thus, exogenous IAA can promote the growth of grape and its uptake of Se. Future studies should focus on the mechanism of translocation of Se to the aboveground parts of grape.

### Electronic supplementary material

Below is the link to the electronic supplementary material.

Supplementary Material 1

## Data Availability

All data generated or analyzed during this study are included in this published article.
